# Relation of Prenatal Methylmercury Exposure from Environmental Sources to Childhood IQ

**DOI:** 10.1289/ehp.1408554

**Published:** 2015-03-10

**Authors:** Joseph L. Jacobson, Gina Muckle, Pierre Ayotte, Éric Dewailly, Sandra W. Jacobson

**Affiliations:** 1 Department of Psychiatry and Behavioral Neurosciences, Wayne State University School of Medicine, Detroit, Michigan, USA; 2 Université Laval and Centre de Recherche du CHU de Québec, Québec, Québec, Canada

## Abstract

**Background:**

Although prenatal methylmercury exposure has been linked to poorer intellectual function in several studies, data from two major prospective, longitudinal studies yielded contradictory results. Associations with cognitive deficits were reported in a Faroe Islands cohort, but few were found in a study in the Seychelles Islands. It has been suggested that co-exposure to another contaminant, polychlorinated biphenyls (PCBs), may be responsible for the positive findings in the former study and that co-exposure to nutrients in methylmercury-contaminated fish may have obscured and/or protected against adverse effects in the latter.

**Objectives:**

We aimed to determine the degree to which co-exposure to PCBs may account for the adverse effects of methylmercury and the degree to which co-exposure to docosahexaenoic acid (DHA) may obscure these effects in a sample of Inuit children in Arctic Québec.

**Methods:**

IQ was estimated in 282 school-age children from whom umbilical cord blood samples had been obtained and analyzed for mercury and other environmental exposures.

**Results:**

Prenatal mercury exposure was related to poorer estimated IQ after adjustment for potential confounding variables. The entry of DHA into the model significantly strengthened the association with mercury, supporting the hypothesis that beneficial effects from DHA intake can obscure adverse effects of mercury exposure. Children with cord mercury ≥ 7.5 μg/L were four times as likely to have an IQ score < 80, the clinical cut-off for borderline intellectual disability. Co-exposure to PCBs did not alter the association of mercury with IQ.

**Conclusions:**

To our knowledge, this is the first study to document an association of prenatal mercury exposure with poorer performance on a school-age assessment of IQ, a measure whose relevance for occupational success in adulthood is well established. This association was seen at levels in the range within which many U.S. children of Asian-American background are exposed.

**Citation:**

Jacobson JL, Muckle G, Ayotte P, Dewailly É, Jacobson SW. 2015. Relation of prenatal methylmercury exposure from environmental sources to childhood IQ. Environ Health Perspect 123:827–833; http://dx.doi.org/10.1289/ehp.1408554

## Introduction

The developmental neurotoxicity of methylmercury first became evident in the mid-twentieth century when infants born to women who had eaten heavily contaminated fish in Minimata Bay, Japan (Harada 1995) and bread contaminated with a methylmercury fungicide in Iraq (Amin-Zaki et al. 1974) exhibited central nervous system impairments, including mental retardation, cerebral ataxia, and seizures. In the 1990s, the National Institute of Environmental Health Sciences (NIEHS) funded two prospective, longitudinal studies on lower-level prenatal methylmercury exposure—one in the Faroe Islands in the North Atlantic (Grandjean et al. 1997), the other in the Seychelles Islands in the Indian Ocean (Davidson et al. 1998). The primary source of mercury exposure in the Faroes is pilot whale meat; the primary source in the Seychelles is fish. Although levels of mercury exposure were similar in the two cohorts, and both studies were well designed and well powered, they yielded contradictory findings. The Faroes study found associations with poorer cognitive and fine motor function in childhood and adolescence (Debes et al. 2006; Grandjean et al. 1997); the Seychelles study did not (Davidson et al. 1998; Myers et al. 2003).

Two expert panels were convened to evaluate these contradictory findings—one by the White House Office of Science and Technology Policy (NIEHS 1998), the other by the National Academy of Sciences [National Research Council (NRC) 2000]. Neither panel was able to provide a definitive explanation for the inconsistencies between the two studies’ findings. One hypothesis noted that the Faroes population was also exposed to significant levels of polychlorinated biphenyls (PCBs), persistent synthetic hydrocarbon compounds once used for industrial purposes that have been linked to deficits in cognitive function (Jacobson and Jacobson 1996; Jacobson et al. 1985; Stewart et al. 2008). PCB concentrations are exceedingly low in the Seychelles (Davidson et al. 1998).

An alternative explanation for the apparent absence of adverse effects in the Seychelles study relates to the nutritional benefits of fish in the diet. Many methylmercury-contaminated fish and sea mammal species are also rich in long-chain polyunsaturated fatty acids (LCPUFA), including docosahexaenoic acid (DHA; 22:6n-3), an important constituent of gray matter in cerebral cortex (Ghys et al. 2002) and the photoreceptor outer segment membranes of the retina (Innis 1991); and selenium, which has been found to protect against methylmercury toxicity in laboratory animal studies (Park et al. 1996). The Faroes study found no evidence of protective effects from selenium on the associations of mercury exposure with cognitive function (Choi et al. 2008). However, DHA was not measured in either the Faroes or the original Seychelles study, so it was not possible to test directly whether its presence in methylmercury-contaminated fish may have obscured adverse effects of the mercury exposure on child development.

Associations of prenatal mercury exposure with poorer cognitive function have also been reported in a sample of 6-year-old children in New Zealand exposed at levels similar to those in the Faroes and Seychelles studies (Crump et al. 1998) and at lower levels of exposure in infants and preschool-age children in the United States and Japan (Lederman et al. 2008; Oken et al. 2005; Suzuki et al. 2010). The hypothesis that co-exposure to beneficial nutrients in fish can obscure the adverse effects of mercury exposure was supported by Budtz-Jørgensen et al. (2007), who found stronger associations of mercury with cognitive function in the Faroes’ data when maternal fish consumption during pregnancy was added to the statistical model (see also Oken et al. 2005). Moreover, in the second Seychelles cohort, in which LCPUFA were measured in maternal serum during pregnancy, inclusion of DHA strengthened the association of prenatal mercury with motor function in infancy (Strain et al. 2008), although not with cognitive or motor function at 5 years (Strain et al. 2012).

To our knowledge, none of the studies to date have documented associations of prenatal mercury exposure with IQ in school-age children. The Faroes study, the only previous study to report associations with poorer cognitive function during school age, found deficits in five very different domains—fine motor coordination, sustained attention, working memory, vocabulary, and declarative memory—using a series of narrowly focused neuropsychological tests. The diversity of these domains suggests an adverse effect on overall cognitive function, but the effect sizes within these discrete domains, calculated by the National Academy of Sciences panel (NRC 2000), were small (median, β = –0.075) and may have underestimated the effect size relative to what might be seen on a comprehensive IQ test. IQ scores are of particular interest because their clinical relevance is well established, with moderate correlations (*r* ~ 0.50) between school-age IQ and adult occupational status (Jencks et al. 1972; McCall 1977).

We report findings from a cohort of school-age Inuit children in Arctic Québec who were exposed prenatally to methylmercury at levels similar to those in the Faroes and Seychelles studies. This study was designed to address two hypotheses relating to the inconsistent findings from the previous studies: *a*) that the associations of prenatal methylmercury exposure with cognitive impairment are not attributable to co-exposure to PCBs, and *b*) that co-exposure to DHA and selenium *in utero* may partially obscure adverse effects of methylmercury exposure on cognitive function. To our knowledge, this is the first study to examine effects of prenatal mercury exposure on a measure of school-age IQ in which biomarkers were used to document DHA and selenium exposure.

## Methods

*Participants*. We tested 286 children from 14 Inuit villages in Arctic Québec, from whom umbilical cord blood samples had been obtained under the auspices of the Arctic Cord Blood Monitoring Program (Muckle et al. 1998). Seventy of the mothers had also provided hair samples during pregnancy (Jacobson et al. 2008; Muckle et al. 2001). The Nunavik region of Québec, where these children live, is located north of the 55th parallel, about 1,500 km from Montréal. Children from smaller villages were transported by plane with their mothers to one of the three largest villages for assessment. Written informed consent was obtained from the parent, as was written assent from each child, following procedures approved by the Wayne State University Human Investigation Committee and the Laval University Research Ethics Committee.

*Procedure*. Each child was assessed on seven subtests comprising three of the four index scores from the Wechsler Intelligence Scales for Children, 4th edition (WISC-IV; Wechsler 2003), and two tests of verbal proficiency adapted for Inuit culture. The WISC-IV subtests for Perceptual Reasoning were Block Design, Picture Completion, and Matrix Reasoning; for Working Memory the subtests were Digit Span and Arithmetic; and for Processing Speed the subtests were Coding and Symbol Search. Because the WISC-IV Verbal Comprehension subtests were not considered culturally appropriate for the Inuit, verbal proficiency was assessed on the Boston Naming Test (BNT) (Kaplan et al. 1983) and Verbal Fluency Test (VFT) (Delis et al. 2001). In BNT, the child is asked to name 60 objects represented by line drawings; in VFT, as many items as s/he can from each of three categories (animals, boys’ names, modes of transportation) within 60 sec. We replaced 20 BNT drawings likely to be unfamiliar to Inuit children with objects more familiar in their environment (e.g., a volcano was replaced by an avalanche). The tests were administered in Inuktituk by a retired Inuit teacher with previous training and experience in administering neuropsychological assessments. BNT and VFT scores were standardized to mean ± SD = 10 ± 3.

Estimated IQ was computed by summing standard scores from the seven WISC-IV subtests and the two standardized verbal test scores, after multiplying the verbal scores by 1.5 to weight them equivalently to the three WISC-IV verbal subtests. Estimated Verbal Comprehension Index (VCI) was computed by summing the BNT and VFT standard scores. A validation study, conducted in a Detroit, Michigan, sample, showed that estimated IQ correlated strongly with standard WISC-IV full-scale IQ (intraclass *r* = 0.92), indicating that our estimated IQ measure reflects intellectual competence in the same domains assessed on the standard WISC-IV (see Supplemental Material, “Validation of Our Adaptation of the WISC-IV IQ Test for Inuit Culture”). Estimated IQ is, therefore, henceforth referred to as IQ. Data for four children were excluded—one could not perform several subtests due to severe mental disability; the others refused to cooperate with the testing procedures.

The mother was interviewed regarding demographic background, smoking, alcohol and drug use during pregnancy and whether the child was adopted, a common practice among the Inuit (Silk 1987). Each mother was also administered the Peabody Picture Vocabulary Test (Dunn and Dunn 1981) in English or French and the nonverbal Raven (1996) Progressive Matrices. A 20-mL venous blood sample was obtained from each child immediately following the cognitive assessment.

Mercury in cord blood and maternal hair was determined by cold vapor atomic absorption spectrometry (Pharmacia Model 120); cord lead, by graphite furnace atomic absorption with Zeeman background correction (Perkin Elmer model ZL 4100); selenium, by inductively coupled plasma mass spectrometry (ICP-MS); mercury, lead, and selenium in child blood by ICP-MS. The 14 most prevalent PCB congeners (IUPAC Nos. 28, 52, 99, 101, 105, 118, 128, 138, 153, 156, 170, 180, 183, 187) were measured in purified cord plasma extracts by gas chromatography with electron capture detection and in purified child plasma extracts, by gas chromatography–mass spectrometry, as were 10 organochlorine pesticides (aldrin, α-chlordane, γ-chlordane, *cis*-nonachlor, DDE (dichlorodiphenyldichloroethylene), DDT (dichlorodiphenyltrichloroethane), hexachlorobenzene, oxychlordane, mirex, and *trans*-nonachlor). PCB congener 153, expressed on a lipid basis, was used as an indicator of total PCB exposure because it is the most abundant PCB congener and is highly correlated with the other congeners (Ayotte et al. 2003). Cord and current mercury, PCB-153, lead, and selenium were subjected to log transformation.

Cholesterol and triglycerides were analyzed using a Hitachi 917 Chemistry Analyzer. Total plasma lipids were then estimated from cholesterol and triglyceride levels. Total phospholipids were isolated from the lipid extract by chromatography, and the fatty acid profile, including DHA, was determined by capillary gas–liquid chromatography as described by Jacobson et al. (2008). Limits of detection (LODs) in cord blood were 0.2 μg/L for mercury and lead, 7.9 μg/L for selenium, 0.02 μg/L for all PCB congeners. LODs in child blood were 0.1 μg/L for mercury, 0.02 μg/L for lead, and 7.1 μg/L for selenium, and < 0.05 μg/L for most PCB congeners. A value equal to half the LOD was entered whenever a substance was not detected.

Twelve control variables were assessed: child sex and age at assessment; child adopted (yes/no); child transported to assessment by plane (yes/no); a social environment composite score constructed by averaging four measures after transformation to standard scores—socioeconomic status (SES) (Hollingshead 2011); primary caregiver’s years of education; Peabody vocabulary and nonverbal Raven test scores; parity (measured as a continuous variable); marital status (married/unmarried); mother sufficiently fluent to be interviewed in English or French, an indicator of assimilation to Western culture (yes/no); age of biological mother at child’s birth; and alcohol (yes/no), smoking (cigarettes/day), and marijuana use (days/month) during pregnancy.

*Data analysis*. The relation of each of the six contaminant exposures (cord and current mercury, PCB-153, and lead) to IQ was examined using a series of nested multivariable regression models. The contaminant of interest (e.g., cord mercury) was entered in the first step; the other contaminants, in the second (model 1); nutrient biomarkers (cord and current DHA and selenium), in the third (model 2); the control variables, in the fourth (model 3). At each step, the variables were entered individually in order of the magnitude of their zero-order correlations with IQ and retained in the model only if their entry altered the relation of the initial contaminant to IQ (standardized regression coefficient β) by at least 10% (Greenland and Rothman 1998). An alpha level of 0.05 was required for an inference of statistical significance. The final covariate-adjusted regression models for each of the exposures were checked by plotting predicted values for IQ against residuals. Visual inspection showed no non-normality or heteroscedasticity. Q-Q plots indicated that the residuals were normally distributed in all models. Two tests were used to identify influential points: None of the standardized residuals fell between –3.0 and 3.0, and no cases exceeded the recommended cut-offs for Cook’s distance.

For each contaminant significantly related to IQ in model 3, post hoc analyses were performed to examine the degree to which prenatal exposure to each of the nutrients may partially obscure the negative association of the exposure with IQ. Each nutrient was added to model 1 to determine whether its entry significantly increased the β for the contaminant exposure based on the Clogg et al. (1992) Difference in Coefficients Test. Dose dependence was examined for each contaminant that related significantly to IQ. The contaminant levels were divided into sextiles and examined in relation to IQ using analysis of variance. Fisher’s Least Significance Difference test was used to identify discontinuities in the dose–response relations of the contaminants to IQ. For each contaminant significantly related to IQ in Model 3, synergistic effects of co-exposure to the other contaminants were examined by adding interaction terms to model 3 (e.g., cord mercury × cord PCB153, cord mercury × cord lead). Protective effects of co-exposure to DHA or selenium were also tested by adding interaction terms to model 3.

Additional analyses were conducted to assess potential confounding by the 10 organochlorine pesticides listed above. Each pesticide even weakly correlated with IQ (at *p* < 0.10) was added to each significant contaminant’s model 3 to determine whether its entry altered the association between that contaminant and IQ.

## Results

Sample characteristics are summarized in Table 1. The families were predominantly of lower SES; 44.0% were employed as unskilled or semiskilled laborers; only 2.1% were major business owners or professional. Only 50.7% of the primary caregivers had completed eighth grade, and 2.5% had completed high school. Nevertheless, a large proportion of the children performed well on the IQ test; 67.0% of the IQ scores fell within 1 standard deviation of the mean for the U.S.-normed WISC-IV sample (i.e., 85–115), compared with the 68.0% expected to fall in that range. Based on the cut-offs for the WISC provided by Sattler (1992), one child’s score indicated mental deficiency (< 70), and 14.5% scored in the “borderline” range (70–79), which indicates mild intellectual disability. Table 1 shows the correlations between the control variables and IQ. The strongest association was with social environment, confirming the critical role of SES and maternal intellectual competence in promoting cognitive function and providing convergent validation for use of the WISC-IV with this Inuit sample. IQ was not related to whether the child was transported by plane for testing or was adopted. Intercorrelations among the exposure measures are provided in Supplemental Material, Table S1.

**Table 1:** Sample characteristics and relations of control variables to IQ score.

Characteristic	Total *n*	Mean ± SD	Range	*n* (%)	Relation to IQ score (*r*)
Primary caregiver
SES^*a*^	282	28.4 ± 11.7	8.0–66.0
Years of education	282	8.5 ± 2.5	0.0–16.0
Social environment composite^*b*^	282	–0.02 ± 0.8	–2.5–2.3		0.29**
Marital status (married)	282			124 (44.0)	0.11^#^
Fluent in English or French	282			256 (90.8)	0.10^#^
Pregnancy history
Maternal age at delivery	282	24.0 ± 5.9	15.0–42.0		0.04
Parity	282	2.0 ± 1.8	0.0–9.0		–0.04
Alcohol during pregnancy (yes)	241			70 (29.0)	0.09
Smoking during pregnancy (cigarettes/day)	269	8.7 ± 7.3	0.0–50.0		–0.14*
Marijuana during pregnancy (days/month)	239	0.4 ± 1.0	0.0–3.4		–0.03
Child
Age at assessment	282	11.3 ± 0.8	8.6–14.3		0.08
Sex (female)	282			143 (50.7)	0.23**
Adopted	282			47 (16.7)	–0.06
Transported by plane	282			98 (34.8)	–0.08
WISC-IV scores
Estimated Full Scale IQ score	282	91.9 ± 11.7	61.0–125.0
Perceptual Reasoning Index	282	94.2 ± 11.2	61.0–127.0
Working Memory Index	282	89.5 ± 11.6	56.0–132.0
Processing Speed Index	282	86.6 ± 12.2	59.0–118.0
Estimated Verbal Comprehension Index	276	84.5 ± 15.1	38.8–116.9
Exposure to contaminants
Prenatal
Cord blood mercury (μg/L)	279	21.8 ± 17.5	1.0–99.3
Maternal hair mercury (μg/g)	70	4.9 ± 2.8	1.4–15.1
Cord plasma PCB-153 (ng/g lipid)	278	120.3 ± 95.0	9.7–653.6
Cord blood lead (μg/dL)	279	4.8 ± 3.4	0.8–20.9
Current
Blood mercury (μg/L)	278	4.7 ± 4.7	0.1–34.1
Hair mercury (μg/g)	277	6.9 ± 6.5	0.3–45.0
Plasma PCB-153 (ng/g lipid)	276	73.5 ± 82.9	3.5–809.5
Blood lead (μg/dL)	278	2.7 ± 2.3	0.4–12.8
Nutrient intake
Prenatal
Cord plasma DHA (% fatty acids)	274	3.6 ± 1.2	1.1–7.7
Cord blood selenium (μg/L)	262	336.3 ± 181.4	112.1–1579.2
Current
Plasma DHA (% fatty acids)	277	2.4 ± 1.0	0.1–5.5
Blood selenium (μg/L)	278	199.6 ± 91.3	67.9–947.5
^***a***^Hollingshead (2011). ^***b***^Based on socioeconomic status, caregiver years of education, Peabody Picture Vocabulary Test raw score, Raven Progressive Matrices raw score, which were converted to standard scores and averaged. Pearson correlations with IQ were 0.26 for socioeconomic status, 0.12 for caregiver education, 0.32 for Peabody Vocabulary, and 0.19 for the Raven. *p < 0.05. **p < 0.001. ^#^*p *< 0.10.					

Prenatal mercury exposure levels were similar to those reported in the Faroes and Seychelles studies, markedly higher than in southern Québec and other general population samples, but similar to those in infants born to native Chinese mothers in New York City (Lederman et al. 2008; see also Supplemental Material, “Comparison of Exposure Levels with Those Reported in Other Studies”). Cord PCB-153 concentrations were similar to or higher than those in previous U.S. and Dutch studies reporting adverse effects on childhood cognitive function (Jacobson and Jacobson 1996; Patandin et al. 1999; Stewart et al. 2008) but much lower than in the Faroes (Longnecker et al. 2003). Prenatal lead was slightly lower than in two general population U.S. studies (Bellinger et al. 1992; Dietrich et al. 1987). Childhood mercury and lead levels were low, but PCB levels during childhood were among the highest reported to date (see Supplemental Material, “Comparison of Exposure Levels with Those Reported in Other Studies”).

Table 2 presents the regression analysis results relating the contaminant exposures to IQ. Two of the exposures—prenatal mercury and prenatal lead—were associated with lower IQ scores after adjustment for exposures to other contaminants (model 1), nutrient biomarkers (model 2), and the other potential confounding variables (model 3). By contrast, neither prenatal PCB-153 nor any of the current contaminant exposure measures were related to IQ.

**Table 2:** Relations of contaminant exposures to IQ.

Exposure	*n*	Pearson *r *(*p*-value)	Model 1		Model 2		Model 3	
			β (95% CI)	*p-*Value	β (95% CI)	*p-*Value	β (95% CI)	*p-*Value
Prenatal
Mercury^*a*^	251	–0.19 (0.001)	–0.15 (–0.29, –0.02)	0.025	–0.21 (–0.36, –0.07)	0.004	–0.17 (–0.31, –0.02)	0.021
PCB-153^*b*^	241	–0.07 (0.134)	0.03 (–0.11, 0.17)	0.665	0.00 (–0.14, 0.15)	0.971	–0.04 (–0.17, 0.10)	0.607
Lead^*c*^	268	–0.19 (0.001)	–0.11 (–0.24, 0.01)	0.077	–0.13 (–0.25, –0.01)	0.037	–0.13 (–0.24, –0.01)	0.038
Current
Mercury^*d*^	240	–0.07 (0.130)	0.04 (–0.11, 0.19)	0.570	0.11 (–0.08, 0.30)	0.274	0.16 (–0.03, 0.36)	0.094
PCB-153^*e*^	214	–0.09 (0.091)	0.03 (–0.12, 0.19)	0.667	0.04 (–0.13, 0.20)	0.664	0.03 (–0.13, 0.19)	0.701
Lead^*f*^	268	–0.17 (0.003)	–0.14 (–0.26, –0.03)	0.016	–0.16 (–0.27, –0.04)	0.007	–0.05 (–0.17, 0.07)	0.429
Covariates for each model were selected based on a 10% change in the model coefficient, as described in the “Methods.” Model 1 covariates are for the selected contaminants only, model 2 includes model 1 covariates plus selected nutrient biomarkers, and model 3 includes model 2 covariates plus other potential confounders.^***a***^Covariates: model 1 = cord lead, current PCB-153; model 2 = + cord selenium, cord DHA; model 3 = + social environment. ^***b***^Covariates: model 1 = cord mercury, cord lead, current PCB-153; model 2 = + cord selenium, cord DHA, current selenium; model 3 = + sex, social environment, marital status, maternal smoking during pregnancy. ^***c***^Covariates: model 1 = cord mercury, current lead; model 2 = + cord DHA; model 3 = + sex, social environment. ^***d***^Covariates: model 1 = cord mercury, current PCB-153, current lead; model 2 = + cord selenium, cord DHA, current selenium, current DHA; model 3 = + sex, age at testing, social environment, maternal smoking during pregnancy. ^***e***^Covariates: model 1 = cord mercury, cord lead, cord PCB-153, current lead; model 2 = + cord selenium, cord DHA, current selenium, current DHA ; model 3 = + sex, age at testing, social environment, marital status, maternal alcohol during pregnancy. ^***f***^Covariates: model 1 = cord mercury; model 2 = + cord DHA; model 3 = + sex, age at testing, social environment, marital status.								

The initial adjustment for the other contaminants weakened the association of mercury with IQ (model 1). However, the association with mercury became stronger when cord DHA and selenium were entered in model 2, indicating that the negative association between prenatal mercury and IQ was biased toward the null due to negative confounding. Post hoc analysis showed that the addition of DHA to model 1 significantly increased the β for cord mercury from –0.15 to –0.19, *t*(250) = 2.03, *p* < 0.05, providing support for the hypothesis that the beneficial effect of higher prenatal DHA obscured the adverse effect of prenatal mercury exposure. [Similarly, the entry of cord mercury in a regression of IQ on DHA and the other contaminants significantly increased the β for DHA from 0.17 to 0.20, *t*(250) = 1.93, *p* < 0.05.] The entry of cord DHA to the cord lead regression marginally increased the β for prenatal lead exposure—from –0.11 to –0.13, *t*(267) = 1.86, *p* < 0.10. By contrast, the entry of cord selenium after controlling for the other exposures did not significantly increase the β for cord mercury, *t*(250) = 0.77, *p* > 0.20, or lead, *t*(267) = 0.26, *p* > 0.20.

Verbal Comprehension and Perceptual Reasoning were the WISC-IV domains most sensitive to prenatal mercury exposure [model 3 β = –0.15; 95% confidence interval (CI): –0.30, –0.004 and β = –0.18; 95% CI: –0.30, –0.06, respectively] (see Supplemental Material, Table S2). Working Memory was the most sensitive to prenatal lead (model 3 β = –0.15; 95% CI: –0.27, –0.03).

**Figure 1:**
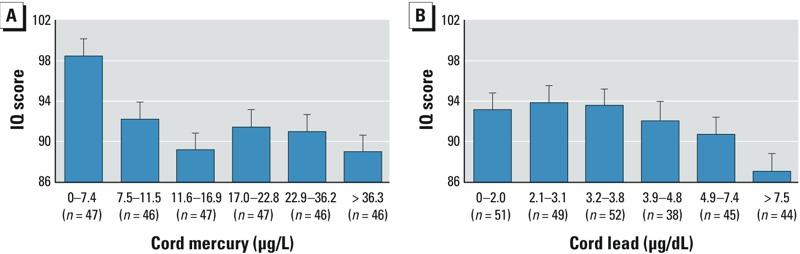
Mean ± SD child IQ scores according to sextiles of (*A*) prenatal mercury and (*B*) prenatal lead. Based on Fisher’s Least Significance Difference tests, estimated mean IQ scores are significantly lower for all sextiles of prenatal mercury relative to the estimated mean for the lowest exposure group (all *p* < 0.005). Estimated mean IQ scores are significantly higher for the first through fourth sextiles of prenatal lead relative to the highest exposure group (all *p* < 0.05).

In an analysis of variance without adjustment for covariates, IQ was significantly lower in all five groups of children with cord mercury concentrations ≥ 7.5 μg/L, compared with those in the least-exposed sextile (Figure 1A). After adjustment for the covariates in Table 2 (cord lead, current PCB-153, cord DHA, cord selenium, social environment), IQ in the least-exposed group (mean + SD = 95.8 + 11.7) was 4.8 points higher than in the other five groups (mean + SD = 91.0 + 11.0), *F*(1,244) = 5.25, *p* < 0.05. By contrast, the association of prenatal lead exposure with lower IQ was clearly evident only in the most heavily exposed children (> 7.5 μg/dL) (Figure 1B). After adjustment for covariates (cord mercury, current lead, cord DHA, sex, social environment), IQ in the highest-exposed sextile (mean + SD = 88.6 + 11.6) was 4.5 points lower than in the four least-exposed groups (mean + SD = 93.1 + 11.1) *F*(1,218) = 4.96, *p* < 0.05.

The functional significance of these deficits was evaluated by examining the incidence of IQ scores < 80, which is considered the upper bound cut-off for borderline intellectual disability (Wechsler 2003). In chi-square analyses without adjustment for covariates, children with higher cord mercury (> 7.5 μg/L) were four times more likely to score in that range; 45 (17.2%) of the more heavily exposed children scored < 80, compared with only 2 (4.3%) of the least-exposed group, χ^2^(1) = 5.15, *p* = 0.013. For prenatal lead exposure, the most heavily exposed group was twice as likely to score in the borderline range—13 (29.5%) compared with 24 (12.6%) of those in the four least-exposed groups, χ^2^(1) = 7.68, *p* = 0.008.

None of the interaction terms evaluating synergistic/protective effects of DHA, selenium, and the other exposures on the relation of cord mercury or lead to IQ were significant (all *p* > 0.20). Among the pesticides weakly related to IQ (at *p* < 0.10), none altered the association between prenatal mercury and IQ when added to model 3 of the regression analysis, but the association of prenatal lead with IQ was reduced slightly by the addition of two cord pesticide measures—DDT and oxychlordane—from β = –0.13 (95% CI = –0.24, –0.01), *p* < 0.05, to β = –0.11 (95% CI = –0.22, 0.01), *p* = 0.078.

## Discussion

The neurotoxicity of exposure to high doses of methylmercury was clearly established in the Minimata Bay (Harada 1995) and Iraq (Amin-Zaki et al. 1974) studies. However, the degree to which lower level prenatal exposure is associated with long-term effects on cognitive function has continued to be uncertain due to the contradictory findings from the Faroes and Seychelles studies. Cognitive deficits have been reported in several studies of infants and young children exposed prenatally to mercury (e.g., Crump et al. 1998; Oken et al. 2005), including our own infants in Nunavik (Boucher et al. 2014). However, to our knowledge, this is the first study to examine effects on cognitive function at school age since the Faroes and Seychelles studies were conducted in the 1990s. As in the Faroes, prenatal mercury was associated with poorer performance in diverse domains of intellectual function, including both Perceptual Reasoning and Verbal Comprehension. Although the IQ scores of the more heavily exposed children (cord Hg > 7.5 μg/L) were only 4.8 points lower on average than the less heavily exposed children, this finding is clinically meaningful in that prenatal mercury was associated with a 3-fold increase in performance below the established cut-off for borderline intellectual disability. It is noteworthy that the association with IQ was seen at exposure levels in the range of those observed in New York City preschool-age children born to native Chinese mothers (mean + SD = 17.0 + 13.0 μg/L) (Lederman et al. 2008).

*The influence of DHA*. This study is, to our knowledge, the first to provide evidence that adverse effects of prenatal mercury exposure on school-age cognitive function can be more difficult to detect in children who are also exposed prenatally to DHA. The significant increase in the magnitude of the estimate for cord mercury after DHA was added to model 1 indicates that the beneficial effect of higher prenatal DHA intake statistically suppressed (i.e., tended to obscure) the adverse effect of the prenatal mercury exposure. By contrast, the addition of selenium did not significantly alter the association with mercury.

Given that none of the interaction terms even approach statistical significance, there is no indication that DHA serves as an antagonist that directly blocks the adverse effects of mercury. The associations of IQ with mercury and DHA are independent. Mercury impairs cognitive development, DHA facilitates it (Colombo et al. 2004; Helland et al. 2003; Jacobson et al. 2008), and the estimate for each is increased by statistical adjustment for the other. This finding of negative confounding is consistent with the hypothesis that the association with mercury is obscured in children simultaneously exposed to DHA because the DHA helps improve their cognitive performance. Similarly, the beneficial effects of DHA are less evident when there is co-exposure to a contaminant that impairs the child’s performance (Jacobson et al. 2008). Our finding that none of the interactions with selenium were significant is consistent with the failure to find a protective effect from selenium in the Faroe Islands study (Choi et al. 2008).

*Prenatal exposure to PCBs and lead*. An association of mercury with IQ was clearly seen after controlling for PCB-153 exposure, and there was no evidence of intensification of this association at higher levels of PCB exposure (cord mercury × cord PCB-153 interaction β = 0.21 95% CI: –0.79, 1.21, *p* = 0.682). Thus, these data provide new evidence that co-exposure to PCBs does not account for the associations of mercury with cognitive performance that were seen in the Faroes but not in the Seychelles. These data are consistent with findings from the Faroes study that the associations with mercury remained significant after controlling for PCB exposure (Grandjean et al. 1997) and that in a stratified analysis the associations with mercury were actually stronger in the children with the lowest PCB exposure levels (Budtz-Jørgensen et al. 1999).

Our data did not confirm the associations of prenatal PCB exposure with lower IQ found in U.S. studies in Michigan (Jacobson and Jacobson 1996) and New York (Stewart et al. 2008). PCBs are complex mixtures of dozens of congeners, each with its own unique molecular structure and potentially very different toxic effects. Comparisons of the congener profile in the current sample with that seen in the Michigan cohort indicate that the potentially more neurotoxic lower chlorinated congeners (Hansen 1998) constituted a much smaller proportion of the PCB mixture to which these Inuit children were exposed *in utero,* when compared with the Michigan children (see Supplemental Material, “Comparisons of PCB Congener Profiles in Michigan and Arctic Québec”). Thus, the failure to confirm previous evidence of PCB neurotoxicity in the current study is likely attributable to exposure to a markedly less neurotoxic PCB congener mix.

Prenatal lead exposure was associated with lower IQ. By contrast to mercury, however, the association with lead was clearly evident only in the most heavily exposed children. Most of the previous studies that have examined long-term effects of prenatal lead have not found associations with IQ (e.g., Bellinger et al. 1992; Dietrich et al. 1987; McMichael et al. 1988). The sole exceptions are the Wasserman et al. (2000) Yugoslavia study (M cord lead = 12.0 μg/dL) and a Mexico City study (mean maternal blood lead during pregnancy = 8.0 μg/dL; Schnaas et al. 2006). Thus, by contrast to postnatal lead exposure, where associations with lower IQ are seen at very low levels (3–5 μg/dL) (Canfield et al. 2003; Chiodo et al. 2004), the threshold for an adverse IQ effect from prenatal lead appears to be somewhat higher.

The principal source of lead exposure in the Inuit population is lead pellets that are used in hunting birds and small mammals, disintegrate in the animal on impact, and are ingested together with the meat (Lévesque et al. 2003). Lead body burdens declined substantially in Nunavik after lead shot was banned in 1998 (Dallaire et al. 2003). Our finding that negative confounding by DHA did not significantly increase the estimate for prenatal lead exposure may relate to the fact that the lead and DHA exposures come from different sources (*r* = 0.15, *p* < 0.05). The correlation between mercury and DHA is also modest (*r* = 0.24, *p* < 0.001); although both are found in fish, much of the mercury exposure in Nunavik comes from beluga whale meat (Lemire et al. 2015), which is low in DHA. Similarly, pilot whale is the principal source of mercury in the Faroes. In the New Zealand study (Crump et al. 1998), which also reported associations of prenatal mercury with poorer child cognitive function, much of the exposure was from shark, which is also low in DHA. In the Seychelles, by contrast, where local fish provide the principal exposures to both mercury and DHA, the adverse effects of mercury have proven more difficult to detect.

*Associations with postnatal contaminants exposures*. In contrast with the prenatal exposures, none of the postnatal exposures, measured in terms of the child’s current body burden, were significantly associated with IQ after adjustment for confounders (Table 2). Child mercury levels were substantially lower than the cord blood levels, presumably because mercury is actively transported across the placenta (Stern and Smith 2003) and consumption of traditional Inuit food declined during the decade following the birth of this cohort (Dewailly et al. 2007). Nevertheless, child blood mercury levels were substantially higher than in the United States (mean = 0.7 μg/L) (Centers for Disease Control and Prevention 2005). Despite these higher exposures, as in the Faroes (Debes et al. 2006; Grandjean et al. 1997), no associations were seen in relation to postnatal mercury exposure. Similarly, although the Nunavik children’s current PCB body burdens (mean = 0.4 μg/L) were also substantially higher than in the United States (0.1 μg/L) (see Supplemental Material, “Comparison of Exposure Levels with Those Reported in Other Studies”), current PCB level was not related to IQ (Table 2). This finding from this cohort, with the highest postnatal PCB exposure reported to date, is consistent with the lack of adverse postnatal effects reported in previous studies (Jacobson and Jacobson 1996; Jacobson et al. 1985; Patandin et al. 1999; Rogan et al. 1986; but see also Walkowiak et al. 2001).

In contrast with mercury and PCBs, child blood lead levels were lower in this cohort than in most U.S. studies (e.g., Canfield et al. 2003; Chiodo et al. 2004). Consistent with reports of poorer cognitive function at increasingly lower levels of postnatal lead exposure (Lanphear et al. 2005), child blood lead was associated with lower IQ after adjustment for the other contaminants and nutrients (model 2, Table 2). This association was no longer significant in model 3, however, due to confounding particularly with social environment and sex. When the potential confounders were entered in model 3 in order of the magnitude of their zero-order correlations with IQ, the entry of social environment reduced the estimate for current lead from –0.159, *p =* 0.007, to –0.117, *p =* 0.045, and the addition of sex reduced it further to –0.052, *p =* 0.388.

*Limitations*. The WISC-IV may be culturally biased because the materials were developed for children in the United States; for example, in this study it was necessary to use different subtests to assess the Verbal Comprehension domain. Nevertheless, the data from our validation study in a sample of Detroit children, in which we administered both the adapted Inuit and standard versions of the WISC-IV, suggest that both approaches yield similar IQ scores (see Supplemental Material, “Validation of Our Adaptation of the WISC-IV IQ Test for Inuit Culture”). A second potential limitation relates to the generalizability of the findings given that the data were obtained from a unique genetic and cultural ethnic group. However, they are consistent with Grandjean et al.’s (1997) findings with Scandanavian Faroese children and Lederman et al.’s (2008) multi-ethnic U.S. cohort study of preschool-age children. To adjust for potential confounding, we assessed a broad range of control variables, including maternal alcohol use and smoking during pregnancy, several measures indicative of the quality of intellectual stimulation provided by the parents (SES and primary caregiver’s educational attainment and Peabody vocabulary and nonverbal Raven test scores), and pre- and postnatal exposure to other environmental contaminants. Nevertheless, we recognize that in a correlational study it is never possible to assess all extraneous variables that might be correlated with exposure and provide alternative explanations for observed effects.

## Conclusions

To our knowledge, this is the first study to confirm the associations of prenatal mercury exposure with poorer cognitive function in school-age children reported in the Faroe Islands, and the first to report an association with school-age IQ. This association is clinically meaningful in that the more heavily exposed children had a 3-fold increased likelihood of falling within the borderline range for intellectual disability on school-age IQ, a measure that is predictive of occupational success in adulthood. To our knowledge, this is also the first study to provide evidence of negative confounding by prenatal DHA, which partially obscured the association of prenatal mercury with lower IQ. These data, therefore, support the hypothesis that the failure to detect adverse effects in the Seychelles is attributable, in part, to high levels of DHA in the fish that provide the principal source of the mercury exposure in that population. Thus, these data add to the growing body of evidence indicating that prenatal methylmercury exposure from environmental sources is teratogenic, and indicate that it is associated with clinically meaningful impairment in overall cognitive function at levels of exposure within the range found in the general U.S. population.

## Supplemental Material

(257 KB) PDF
