# Supplementary material for: Relation of Prenatal Methylmercury Exposure from Environmental Sources to Childhood IQ
Source: Environ Health Perspect. 2015 Mar 10;123(8):827–33. doi: 10.1289/ehp.1408554 (PMC4529008; doi:10.1289/ehp.1408554)
Supplement: (257 KB) PDF [file ehp.1408554.s001.acco.pdf]

Environ Health Perspect
doi: 10.1289/ehp.1408554

Note to Readers: ***EHP*** **strives to ensure that all journal content is accessible to all readers. However, some figures and Supplemental Material published in** ***EHP*** **articles may not conform to 508 standards due to the complexity of the information being presented. If you need assistance accessing journal content, please contact ehp508@niehs.nih.gov. Our staff will work with you to assess and meet your accessibility needs within 3 working days.**

Supplemental Material

Relation of Prenatal Methylmercury Exposure from Environmental Sources to Childhood IQ

Joseph L. Jacobson, Gina Muckle, Pierre Ayotte, Éric Dewailly, and Sandra W. Jacobson

Table of Contents

Validation of Our Adaptation of the WISC-IV IQ Test for Inuit Culture

Comparison of Exposure Levels with Those Reported in Other Studies

Comparisons of PCB Congener Profiles in Michigan and Arctic Québec

References

Table S1. Pearson correlations between exposures.

Table S2. Relations of cord mercury and lead to the four WISC-IV index scores.

Validation of Our Adaptation of the WISC-IV IQ Test for Inuit Culture

Validity of the estimated IQ scores was evaluated in 30 children from metropolitan Detroit (17 boys, 13 girls; mean age=10.6 years, SD=1.4), who were administered standard versions of the Wechsler Intelligence Scales for Children, Fourth edition (WISC-IV; Wechsler, 2003), Boston Naming Test (BNT; Kaplan et al. 1983), and Verbal Fluency Test (VFT; Delis et al. 2001) and the Inuit version of the BNT. The two BNT versions were administered on 2 different days at least 6 days apart, with the order counterbalanced. Scores on the two versions were highly correlated, intraclass correlation (ICC)=0.93. Estimated IQ, calculated for the Detroit sample as it had been for the Inuit children, was highly correlated with Full Scale IQ on the standard WISC-IV, ICC=0.92. The inference that the Inuit and standard versions reflect the same domains of cognitive function is supported by the fact that this validity coefficient falls within the range of the test-retest reliability of the WISC-IV (r=0.85-0.92; Sattler and Dumont, 2004). ICC between the standard and estimated Verbal Comprehension Index (VCI) scores for the Detroit sample was 0.73. Estimated VCI scores were normalized for the Inuit sample in relation to the Detroit scores, which were set to mean±SD=100±15.

Comparison of Exposure Levels with Those Reported in Other Studies

Prenatal mercury exposure levels were similar to those reported in the Faroes (maternal hair geometric mean=4.3μg/g; Grandjean et al. 1997) and Seychelles (maternal hair median=5.9μg/g; Myers et al. 1995) studies. In a study of 1107 Southern Québec newborns (Rhainds et al., 1999), cord mercury levels were markedly lower (mean± SD=1.4±1.4μg/L) than in Nunavik. However, the cord mercury levels in our study were similar to those seen in infants born to native Chinese mothers (17.0±13.0μg/L) in New York City (Lederman et al., 2008), and the association with IQ

was seen at exposure levels in Nunavik (Fig. 1) within the range of the exposures in the New York City sample as a whole (7.8±9.7μg/L).

Cord PCB153 concentrations were similar to two studies reporting adverse effects on childhood cognitive function (Jacobson and Jacobson 1996—born 1980-81; Patandin et al., 1999—born 1990-1992), markedly higher than in one study (Stewart et al., 2008—born 1991-1994; median=40ng/g lipids), and much lower than in the Faroes (Longnecker et al. 2003—median=450ng/g lipids). Prenatal lead was slightly lower than in the general U.S. population (6.3-6.8μg/dL; Bellinger et al. 1992; Dietrich et al. 1987).

Current lead was lower than in one U.S. study (Dietrich et al. 1987—Mean=11.8μg/dL) but similar to levels in other general population lead studies. 5-year blood lead available from 92 children in this sample averaged 5.6μg/dL, compared with 5.4-6.3μg/dL in two U.S. studies (Canfield et al. 2003; Chiodo et al. 2004). To compare current PCB levels with those in the Michigan cohort, we adjusted for measurement differences between the wet-weight total PCB measure used in the Michigan study (Jacobson and Jacobson, 1996) and the congener-specific, lipid-adjusted Nunavik measure by multiplying Michigan total PCBs by 0.17, as recommended by Longnecker et al. (2003). 11-year child PCB levels (measured on a wet-weight basis) were substantially higher in this Inuit sample—mean=0.4μg/L, compared with 0.1μg/L in Michigan. Because PCBs are difficult to excrete and remain stored in body fat over extended periods of time, current PCB levels in this sample of children who had eaten substantial quantities of PCB-contaminated food were the highest reported to date. By contrast to PCBs, current mercury levels were relatively low, presumably because the estimated half-life in the body for methylmercury is only 70-80 days (Bernard and Purdue, 1984).

Comparisons of PCB Congener Profiles in Michigan and Arctic Québec

Data from multiple sources suggest that the PCB mixture prevalent in Arctic Québec in 1994-1997 when these children were born was less neurotoxic than the mixture to which Michigan cohort was exposed. The biologically most persistent congeners (138, 153, and 180) are more highly chlorinated and less neurotoxic than many of the lower chlorinated and ortho-chlorinated congeners (Hansen 1998). Dewailly et al. (1993) found a very different congener profile in breast milk samples in Nunavik compared with Southern Canada, with the potentially more neurotoxic lower chlorinated congeners constituting a much smaller proportion of the Nunavik samples. In a comparison of the current sample with the Michigan cohort, we found that the three most persistent congeners were the most prevalent in both cohorts but constituted a markedly larger proportion of the Nunavik PCB mixture—62.5%, compared with 41.4% in Michigan—whereas the Michigan congener profile contained a substantially greater proportion of lower chlorinated congeners. Thus, the failure to confirm previous evidence of PCB neurotoxicity in the current study is likely attributable to exposure to a markedly less neurotoxic PCB congener mix.

We compared profiles from congener specific data obtained from a subsample of the Michigan cohort (Jacobson et al. 1989) to the cord blood samples from the current study. In each cohort, the 14 most prevalent congeners were measured. However, only 8 of the same congeners were among the most prevalent in both cohorts. In Michigan, 5 of the 6 congeners not measured in Nunavik were lower chlorinated (IUPAC Nos. 31 or lower), whereas in Nunavik all of the congeners not detected in Michigan were higher chlorinated (IUPAC Nos. 52 or higher). The potentially neurotoxic lower chlorinated congeners constituted 6.7% of the Michigan samples, compared with only 1.4% of the samples from Nunavik.

References

Bellinger DC, Stiles KM, Needleman HL. 1992. Low-level lead exposure, intelligence and academic achievement: a long-term follow-up study. Pediatrics 90:855-861.

Bernard SR, Purdue P. 1984. Metabolic models for methyl and inorganic mercury. Health Phys 46: 695-699.

Canfield RL, Henderson CR, Cory-Slechta DA, Cox C, Jusko TA, Lanphear BP. 2003. Intellectual impairment in children with blood lead concentrations below 10 microg per deciliter. N Engl J Med 348:1517-1526.

Chiodo LM, Jacobson SW, Jacobson JL. 2004. Neurodevelopmental effects of postnatal lead exposure at very low levels. Neurotoxicol Teratol 26:359-371.

Delis DC, Kaplan E, Kramer J. 2001. Delis-Kaplan Executive Function Scale. San Antonio, TX: The Psychological Corporation.

Dewailly E, Ayotte P, Bruneau S, Laliberte C, Muir DC, Norstrom RJ. 1993. Inuit exposure to organochlorines through the aquatic food chain in arctic quebec. Environ Health Perspect 101**:**618-620.

Dietrich KN, Krafft KM, Bornschein RL, Hammond PB, Berger O, Succop PA, et al. 1987. Low-level fetal lead exposure effect on neurobehavioral development in early infancy. Pediatrics 80:721-730.

Grandjean P, Weihe P, White RF, Debes F, Araki S, Yokoyama K, et al. 1997. Cognitive deficit in 7-year-old children with prenatal exposure to methylmercury. Neurotoxicol Teratol 19:417-428.

Hansen LG. 1998. Stepping backward to improve assessment of PCB congener toxicities. Environ Health Perspect 106:171-189.

Jacobson JL, Humphrey HE, Jacobson SW, Schantz SL, Mullin MD, Welch R. 1989. Determinants of polychlorinated biphenyls (PCBs), polybrominated biphenyls (PBBs), and dichlorodiphenyl trichloroethane (DDT) levels in the sera of young children. Am J Public Health 79:1401-1404.

Jacobson JL, Jacobson SW. 1996. Intellectual impairment in children exposed to polychlorinated biphenyls in utero. N Engl J Med 335:783-789.

Kaplan EF, Goodglass H, Weintraub S. 1983. The Boston Naming Test, 2nd ed. Philadelphia:Lea & Febriger.

Lederman SA, Jones RL, Caldwell KL, Rauh V, Sheets SE, Tang D. et al. 2008. Relation between cord blood mercury levels and early child development in a World Trade Center cohort. Environ Health Perspect 116:1085-1091.

Longnecker MP, Wolff MS, Gladen BC, Brock JW, Grandjean P, Jacobson JL, et al. 2003. Comparison of polychlorinated biphenyl levels across studies of human neurodevelopment. Environ Health Perspect 111:65-70.

Myers GJ, Davidson PW, Cox C, Shamlaye CF, Tanner MA, Choisy O, et al. 1995. Neurodevelopmental outcomes of Seychellois children sixty-six months after in utero exposure to methylmercury from a maternal fish diet: pilot study. Neurotoxicol 16:639-652.

Patandin S, Lanting CI, Mulder PG, Boersma ER, Sauer PJ, Weisglas-Kuperus N. 1999. Effects of environmental exposure to polychlorinated biphenyls and dioxins on cognitive abilities in Dutch children at 42 months of age. J Pediatr 134:33-41.

Rhainds M, Levallois P, Dewailly É, Ayotte P. 1999. Lead, mercury and organochlorine compound levels in cord blood in Québec, Canada. Arch Environ Health 54:40–47.

Sattler JM, Dumont R. 2004. Assessment of Children: WISC-IV and WPPSI-III Supplement. La Mesa, CA: Jerome M. Sattler, Publisher, Inc.

Stewart PW, Lonky E, Reihman J, Pagano J, Gump BB, Darvill T. 2008. The relationship between prenatal PCB exposure and intelligence (IQ) in 9-year-old children. Environ Health Perspect 116:1416-1422.

Wechsler D. 2003. Wechsler Intelligence Scale for Children--4th Edition (WISC-IV). San Antonio, TX:Harcourt Assessment.

Table S1. Pearson correlations between exposures.

Exposure	Prenatal Mercury	Prenatal PCB153	Prenatal Lead	Prenatal Selenium	Prenatal DHA	Current Mercury	Current PCB153	Current Lead	Current Selenium	Current DHA
Prenatal Mercury	1.0									
Prenatal PCB153	0.38***	1.0								
Prenatal Lead	0.30***	0.23***	1.0							
Prenatal Selenium	0.43***	0.24***	0.15*	1.0						
Prenatal DHA	0.24***	0.21***	0.15*	0.12†	1.0					
Current Mercury	0.42***	0.23***	0.15*	0.19**	0.16**	1.0				
Current PCB153	0.38***	0.43***	0.23***	0.21***	0.18**	0.51***	1.0			
Current Lead	0.12†	0.04	0.16**	-0.04	0.10	0.21***	0.28***	1.0		
Current Selenium	0.18**	0.15*	0.08	0.21***	0.10	0.59***	0.36***	0.21***	1.0	
Current DHA	0.07	0.11†	-0.05	0.08	0.32***	0.39***	0.21***	-0.00	0.17**	1.0

Values are Pearson *r*.

†$p < .10$ *$p < .05$ **$p < .01$ ***$p < .001$

Table S2. Relations of cord mercury and lead to the four WISC-IV index scores.

	N	Pearson *r* (*p*-value)	Model 1 β (95% CI)	*p*	Model 2 β (95% CI)	*p*	Model 3 β (95% CI)	*p*
**Cord Mercury**								
Estimated Verbal Comprehension[a]	253	-0.16 (0.007)	-0.10 (-0.24, -0.04)	0.144	-0.18 (-0.33, -0.03)	0.015	-0.15 (-0.30, -0.004)	0.042
Perceptual Reasoning[b]	271	-0.18 (0.001)	-0.18 (-0.30, -0.07)	0.002	-0.23 (-0.34, -0.11)	<0.001	-0.18 (-0.30, -0.06)	0.004
Working Memory[c]	251	-0.11 (0.037)	-0.13 (-0.27, 0.02)	0.080	-0.12 (-0.28, 0.04)	0.133	-0.06 (-0.22, 0.09)	0.419
Processing Speed[d]	259	-0.11 (0.039)	-0.11 (-0.24, 0.03)	0.125	-0.12 (-0.27, 0.03)	0.108	-0.07 (-0.21, 0.07)	0.320
**Cord Lead**								
Estimated Verbal Comprehension[e]	268	-0.13 (0.018)	-0.07 (-0.20, 0.06)	0.272	-0.09 (-0.21, 0.04)	0.164	-0.09 (-0.20, 0.03)	0.158
Perceptual Reasoning[f]	271	-0.13 (0.020)	-0.08 (-0.20, 0.05)	0.229	-0.09 (-0.21, 0.03)	0.141	-0.07 (-0.19, 0.05)	0.276
Working Memory[g]	271	-0.16 (0.003)	-0.14 (-0.26, -0.02)	0.027	-0.16 (-0.27, -0.03)	0.013	-0.13 (-0.26, -0.01)	0.033
Processing Speed[h]	275	-0.13 (0.016)	-0.07 (-0.19, 0.05)	0.263	-0.07 (-0.19, 0.05)	0.263	-0.10 (-0.21, 0.02)	0.106

Covariates for each model were selected based on a 10% change in the model coefficient, as described in the Methods. Model 1 covariates are selected contaminants only, Model 2 includes Model 1 covariates plus selected nutrient biomarkers, Model 3 includes Model 2 covariates plus other potential confounders.

[a]Covariates: Model 1 = current mercury, cord lead; Model 2 = + cord selenium, cord DHA; Model 3 = + social environment. [b]Covariates: Model 1 = none; Model 2 = + cord DHA; Model 3 = + social environment. [c]Covariates: Model 1 = cord mercury, cord PCB-153, current mercury; Model 2 = + cord DHA, current DHA, cord selenium, current selenium; Model 3 = + sex, social environment. [d]Covariates: Model 1 = cord lead, current lead, current mercury; Model 2 = + cord selenium; Model 3 = + sex, social environment, marital status. [e]Covariates: Model 1 = cord mercury, current lead; Model 2 = + cord DHA; Model 3 = + social environment, age at testing. [f]Covariates: Model 1 = cord mercury; Model 2 = + cord DHA; Model 3 = + social environment. [g]Covariates: Model 1 = cord mercury; Model 2 = + cord DHA; Model 3 = + sex, social environment. [h]Covariates: Model 1 = current lead, cord mercury; Model 2 = + none; Model 3 = + sex, marital status.
